# Psychosocial Factors Involved in Genetic Testing for Rare Diseases: A Scoping Review

**DOI:** 10.3390/genes16060614

**Published:** 2025-05-22

**Authors:** Samantha Strasser, Isabella R. McDonald, Melissa K. Uveges, Sharlene Hesse-Biber, Jordan Keels, Neil Smith, Andrew A. Dwyer

**Affiliations:** 1 Global Public Health and the Common Good, Boston College, Chestnut Hill, MA 02467, USA; strasssa@bc.edu; 2 William F. Connell School of Nursing, Boston College, Chestnut Hill, MA 02467, USAuveges@bc.edu (M.K.U.); keelsj@bc.edu (J.K.); 3 Department of Sociology, Boston College, Chestnut Hill, MA 02467, USA; hesse@bc.edu; 4 “I Am HH” Patient Organization, Dallas, TX 75238, USA; 5 P50 Massachusetts General Hospital—Harvard Center for Reproductive Medicine, Boston, MA 02114, USA

**Keywords:** coping, decision-making, genetic counseling, genetic testing, psychosocial factors, person-centered care, rare disease

## Abstract

**Background/Objectives**: Rare diseases are predominantly genetic in etiology and characterized by a prolonged ‘diagnostic odyssey’. Advances in genetic testing (GT) have helped shorten the time to diagnosis for rare/undiagnosed conditions. We aimed to synthesize the evidence on psychosocial factors related to GT for rare diseases to inform more person-centered approaches to care. **Methods**: We conducted a systematic literature search in six databases using structured terms (September 2024). Retrieved articles underwent independent dual review. Data were extracted and collated in tables for analysis. Thematic analysis was used to identify promoters/barriers to GT for patients and families. Findings were validated by a patient advocate and were reported using PRISMA-ScR guidelines. Synthesized findings were mapped to the Theory of Planned Behavior to inform intervention development. **Results**: Of 1730 retrieved articles, 32 were included for data extraction/synthesis. Studies employed qualitative (n = 19), quantitative (n = 10), and mixed-methods (n = 3) approaches. Nearly all (29/32, 91%) were non-interventional, reporting on decision-making cognitions/processes (19/32, 59%), attitudes/preferences (15/32, 47%), psychosocial impact (6/32, 19%), and knowledge/awareness (4/32, 8%) of pre-conception/prenatal/diagnostic GT and carrier screening. Promoters included understanding GT, ending the diagnostic odyssey, actionable outcomes, personal/family history, altruism, and reproductive decision-making. Barriers included logistical (e.g., distance, cost), psychological burden, perceived lack of benefit, and discrimination/social stigma concerns. **Conclusions**: Some psychosocial factors related to GT for rare diseases overlap with those in literature on GT for common conditions. Identified factors represent targets for theory-informed, person-centered interventions to support high-quality GT decisions that are informed and aligned with patient/family values and preferences.

## 1. Introduction

Rare diseases are individually infrequent, yet there are ~7000 rare diseases, affecting an estimated 25–30 million Americans, 36 million Europeans, and between 263–446 million individuals globally [1,2]. Rare diseases are often overlooked because of their low frequency and are poorly understood due to high medical complexity. Notably, limited knowledge of rare disease contributes to delays in diagnosis—termed the ‘diagnostic odyssey’. Patients with a rare disease typically wait five to seven years to receive a correct diagnosis, and more than 40% are misdiagnosed along the way [1]. Timely diagnosis is crucial for initiating disease management treatments/therapies that support improved health outcomes and quality of life. Data show earlier diagnosis is associated with fewer hospitalizations, decreased healthcare costs, and less financial hardship for families [3].

Importantly, approximately 80% of rare diseases are genetic in etiology [3]. Advances in genetic testing (GT), particularly next-generation sequencing technologies (e.g., whole-exome and whole-genome sequencing), have improved the speed and accuracy of rare disease diagnosis [4,5,6]. As such, GT is a powerful tool for shortening the ‘diagnostic odyssey’ and helping to alleviate some of the financial and psychosocial burdens associated with undiagnosed conditions [6,7]. While the technological aspects of GT have advanced, less is known about the psychosocial factors that influence individuals’ (i.e., patients and families) decisions to pursue testing. Psychosocial factors encompass psychological, emotional, sociocultural, and systemic influences that shape decision-making. Psychosocial factors related to GT have been explored in the context of pre-implantation testing [8], pediatric settings [9], and for common (i.e., Tier 1) inherited cancer syndromes [10,11,12]. To date, relatively little is known about the psychosocial factors involved in GT decision-making in the context of rare diseases.

The primary aim of this scoping review is to explore the psychosocial factors related to GT in rare disease literature. Patients and families affected by rare diseases face unique challenges, including health disparities [13]. Understanding the human factor, as well as promoters/barriers to GT for rare diseases, is an important first step for developing more person-centered approaches to GT for rare diseases. Current recommendations for developing complex interventions such as decisional support involve utilizing a theoretical framework to guide intervention development [14]. As such, the secondary aim of this work is to map the synthesized findings onto the Theory of Planned Behavior [15] to identify theory-informed targets for tailored interventions supporting high-quality GT decisions (i.e., informed and aligned with values and preferences) [16].

## 2. Materials and Methods

We conducted a structured, systematic literature search and scoping review to explore what is known about psychosocial factors influencing patient/family decisions for GT for rare disorders. The secondary aim was to synthesize the findings and map them onto the Theory of Planned Behavior to inform future intervention development. The work was exempt from IRB review, and no formal protocol was registered for the scoping review.

We followed the Arksey and O’Malley framework for scoping reviews [17,18] that consists of six sequential steps: (1) identifying the research question; (2) identifying relevant literature; (3) selecting studies; (4) data charting; (5) collating, summarizing, and reporting results; and (6) consultation. Findings were reported using Preferred Reporting Items for Systematic Reviews and Meta-Analyses Extension for Scoping Reviews (PRISMA-SR) guidelines.

### 2.1. Identifying the Research Question

The primary research question was: “What is known about psychosocial factors in GT for rare conditions?” Rare diseases were defined according to the 1984 Orphan Drug Act that defines a rare disease/disorder in the United States (U.S.) as a condition affecting fewer than 250,000 individuals [19]. In Europe, it is defined as 1 or fewer in 2000 people [19]. The secondary research question was “To what extent do identified psychosocial factors align with Azjen’s Theory of Planned Behavior?” The Theory of Planned Behavior (TPB) is a framework that has been broadly adopted to investigate diverse, wide-ranging health behaviors [15].

### 2.2. Identifying the Relevant Literature

In collaboration with a research librarian, we conducted a systematic literature search (29 September 2024) across six electronic databases—PubMed, Web of Science, Cochrane Library, Sociological Abstracts, Embase, and Journal of Biomedical Informatics. The structured search employed relevant keywords and medical subject headings (MeSH) (Appendix A) to identify relevant published articles. We used Covidence™ 2.0 (Veritas Health Innovation, Melbourne, Australia, www.covidence.org) to store retrieved articles for screening and data extraction.

### 2.3. Selecting the Literature

**Figure 1:**
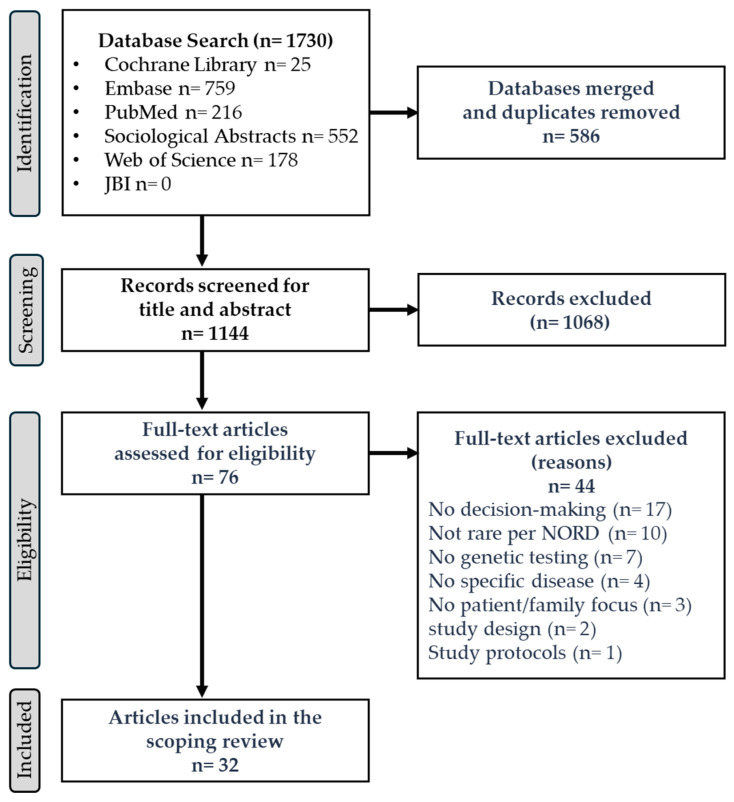
PRISMA Diagram.

Included articles were published in English (1 January 2014 to 1 September 2024). Articles from the past decade were searched to provide a contemporary perspective on the topic. Data-based articles (i.e., quantitative, qualitative, mixed methods, or multiple methods), review articles, and systematic reviews were included. Opinion pieces, study protocols, conference abstracts, and case reports were excluded. Included studies focused on germline GT for rare genetic conditions. Studies focusing on GT for somatic changes, polygenic risk scores, newborn screening, epidemiologic studies, and studies not focusing on patients or families (e.g., healthcare providers) were excluded. All included studies explored factors related to access to genetic counseling/testing, decision-making processes, patient needs, return of results, coping responses, and/or intrafamilial communication of genetic risk. Identified articles were imported into Covidence™ for screening. After removing duplicates, two investigators (S.S. and I.R.M.) independently screened titles and abstracts for relevance. Subsequently, investigators independently conducted full-text reviews. An additional full-text review was conducted to examine disease prevalence to exclude studies not reporting on rare conditions, as defined by the National Organization of Rare Disorders (NORD) database (https://rarediseases.org/rare-diseases/, accessed on 19 May 2025) (Figure 1). Any discrepancies in the screening process were resolved through consensus with a third reviewer (A.A.D.).

### 2.4. Data Charting

Two researchers (S.S. and J.K.) independently extracted the data using a standardized, predetermined data collection form that was created for this scoping review. Extracted information included study title, authors, year of publication, country of origin, journal, measures/tools/questionnaires used, study design, number of participants, participant demographics (including age), inclusion criteria, and key findings. Subtle differences (i.e., key findings) were resolved by discussion. Due to the methodological diversity of the included studies, formal risk of bias assessment was not performed. Results were synthesized using thematic analysis to identify promoters and barriers to GT.

### 2.5. Synthesis of Results

Two researchers (S.S. and J.K.) used thematic analysis [20,21] to identify salient themes. Findings were reviewed and analyzed in an iterative process to identify promoters and barriers to GT. Identified themes were collapsed into concise groups by discussion.

### 2.6. Analysis

Study characteristics were reported using descriptive statistics. Qualitative study findings were reported narratively. We employed thematic analysis [20,21] to identify salient themes emerging from the qualitative study findings. Briefly, thematic analysis involved reading and re-reading the qualitative data to become familiar with the content. Subsequently, initial codes were developed. Codes were subsequently grouped into themes, then reviewed/discussed to determine if they could be collapsed into more concise groupings. After reaching the final identified themes, themes were categorized as promoters or barriers to GT, respectively. Promoters and barriers amenable to intervention were then mapped to the TPB.

### 2.7. Consultation

As part of our patient and public involvement, we had a patient advocate (N.S.) review our findings and synthesis of results. The patient advocate has previously been involved in research examining attitudes towards and experiences with GT in the context of rare diseases. Discussions with the patient advocate elicited feedback to refine the synthesis of findings and underscore salient aspects to inform discussion.

### 2.8. Mapping to the Theory of Planned Behavior

Following synthesis, we mapped identified themes onto the Theory of Planned Behavior (TPB) [15]. Briefly, the TPB posits that intention precedes action. Intentions, in turn, are shaped by attitudes (i.e., perceptions of the behavior being positive or negative), subjective norms (i.e., expectations of family and community healthcare professionals), and perceived behavioral control (i.e., one’s perceived agency and sense that action is up to the individual). The TPB also considers that attitudes, subjective norms, and perceived behavioral control are influenced by beliefs, values, and past experiences. Mapping is intended to inform the development of person-centered interventions to support patients in GT decisions. Decisional support is considered a complex intervention, and the National Health Service recommends incorporating a theoretical framework at the early stages of intervention development [14].

## 3. Results

The systematic, structured literature search identified 1730 articles (Figure 1). After removing duplicates and title/abstract screening, 76 articles underwent full-text review, identifying 32 articles for data extraction and analysis (Table S1).

### 3.1. Study Characteristics

**Figure 2:**
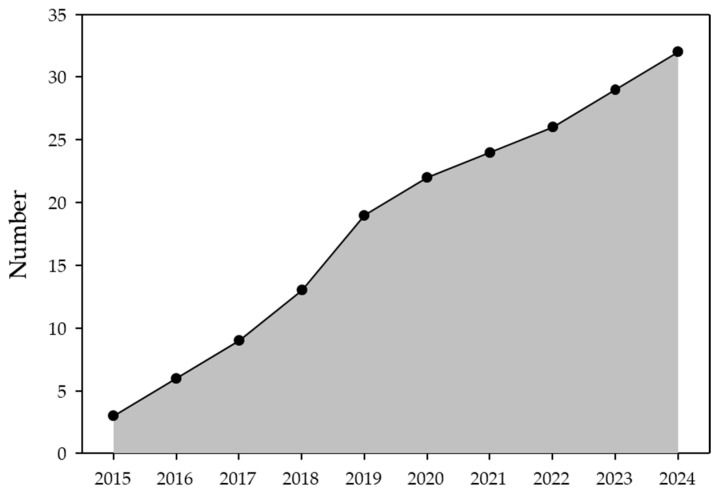
Cumulative publications by year (2014–2024). Publications ranged from 2–6 per year (median = 3): 2014: n = 0, 2015: n = 3, 2016: n = 2, 2017: n = 4, 2018: n = 4, 2019: n = 6, 2020: n = 3, 2021: n = 2, 2022: n = 2, 2023: n = 3, 2024: n = 3.

The number of published articles was quite consistent from 2015 to 2024, ranging from two to six (median: 3) publications per year: 2015 [22,23,24], 2016 [25,26,27], 2017 [28,29,30], 2018 [31,32,33,34], 2019 [35,36,37,38,39,40], 2020 [41,42,43], 2021 [44,45], 2022 [46,47], 2023 [48,49,50], and 2024 [51,52,53] (Figure 2). In total, 20/32 (63%) of the included articles were from Anglophone countries: United States (U.S.) n = 10 [23,25,28,33,34,36,44,45,49,52], Australia n = 5 [24,30,32,41,43], United Kingdom (U.K.) n = 3 [31,42,50], and Canada n = 2 [40,48]. Two articles were from France [26,38] and Germany [29,51], and there was one each from Denmark [47], Portugal [39], Malaysia [22], Turkey [27], and China [37]. Three articles included data from multiple countries (Australia, France, U.K., and U.S.) [35], (U.K. and U.S.) [46], and (Canada and the Philippines) [53].

Non-interventional studies used surveys, interviews, and medical record reviews to examine several factors related to GT, including decision-making cognitions/processes (n = 18) [22,23,24,27,29,30,31,32,33,35,36,38,39,42,44,46,49,52,53], attitudes/preferences (n = 15) [25,26,28,32,34,35,36,37,40,43,46,48,50,51,52], knowledge/awareness (n = 4) [37,42,47,50], and psychosocial impact (n = 6) [30,38,41,45,47,49]. Three articles [30,44,47] reported findings from interventional studies. One was a longitudinal examination of deliberation and decision-making for preconception screening and prenatal GT for Fragile X [30]. Another was a prospective study testing framing effects on GT decisions using hypothetical GT scenarios for a common genetic condition (hereditary breast and ovarian cancer syndrome) and a rare genetic condition (congenital hypogonadotropic hypogonadism) [44]. The third was a mixed-methods study of retinoblastoma survivors who were re-contacted and offered genetic counseling to examine uptake and decision-making [47]. One systematic review on Li–Fraumeni syndrome was included in the analysis [35]. Authors used dual review and employed a quality appraisal tool for the eight articles. Author conclusions were summarized in the data extraction form.

### 3.2. Rare Disorders Under Investigation and Types of Genetic Testing

Included articles examined a range of conditions. Eleven (34%) studies focused on ‘prenatal and reproductive genetics’ [22,24,27,30,31,32,36,42,43,51,52]. Eight (25%) studies explored unspecified ‘rare diseases’ [22,25,26,31,40,42,49,50]. Among articles examining a specific rare disease, the most frequently reported conditions included Li–Fraumeni syndrome (n = 4) [23,35,41,45], rare eye diseases (n = 3) [37,47,53], and two each on Huntington’s disease [29,34], cystic fibrosis [22,24,51], and sickle cell disease [33,52]. Other rare diseases that were investigated in single studies included Fragile X syndrome [30], prion disease [51], Machado–Joseph disease [39], congenital hypogonadotropic hypogonadism [46], and Marfan syndrome [51]. Five studies [22,24,30,48,52] overlapped with the ‘prenatal and reproductive genetics’ category. One interventional study examined hypothetical GT scenarios for congenital hypogonadotropic hypogonadism (rare genetic disorder) and hereditary breast and ovarian cancer syndrome (relatively common genetic disorder) [44].

Studies examined a range of types of GT (Table 1), including diagnostic, predictive (i.e., assessing risk for developing a specific disease in the future), and carrier screening (e.g., Huntington’s disease, Li–Fraumeni syndrome). Two articles reported findings on hypothetical GT scenarios [28,44].

### 3.3. Qualitative Studies (n = 19)

The majority of identified articles (21/32, 69%) reported studies that employed qualitative methods (e.g., semi-structured interviews, focus group discussions [22,23,24,25,26,28,31,34,35,37,39,41,42,44,45,46,49,50,51,52,53]. Thematic and content analyses were commonly employed analytic approaches used to explore cross-sectional patient/family/caregiver perspectives and lived experiences. One study explored participant experiences through the process of GT [25]. None employed formal longitudinal designs. Study sample sizes ranged from 7 [22] to 39 interviews [23]. Participants described GT as valuable for clarifying diagnosis (e.g., shortening the ‘diagnostic odyssey’ and guiding care [25,46]) yet were challenged by logistical barriers, including long wait times [37], limited pre-test counseling [46], and cost [39].

In reproductive and prenatal contexts, participants valued actionable information and emphasized reproductive autonomy [24,26,42,49] while noting the influence of cultural norms/pressures [22] and emotional strain from uncertainty/anxiety [24,49]. Despite psychological distress while awaiting results [31,51], participants often felt GT was empowering and fostered a sense of agency and hope [28,35].

Some adolescents and young adults did not fully understand or were confused by their genetic risk and expressed desire for tailored support and education [34,45]. Several studies underscored the importance of clear communication, calling for enhanced genetic counseling and structured protocols for managing incidental findings [26,52].

### 3.4. Quantitative Studies (n = 10)

Ten (31%) articles reported findings from quantitative studies, half of which employed investigator-developed instruments [27,33,40,43,48]. Five studies employed at least one validated instrument measuring constructs, including decision-making (Satisfaction with Decision Scale, Decision Regret Scale) [30,32,40,44,50], depression/anxiety (Depression and Stress Scale, State-Trait Anxiety Inventory) [30,32], resilience (Brief Resilience Scale) [32], religiosity (Centrality of Religiosity Scale) [43], health/genetic literacy (Newest Vital Sign [44], Genetic Literacy and Comprehension Test) [36], and knowledge/attitudes relating to genomics sequencing (GS) (Knowledge of GS, Attitudes towards GS) [50]. Three studies exploring the GT experiences of pregnant women revealed limited knowledge of GT and poor understanding of risk [27,30,32]. Age, educational attainment, knowledge of GT (e.g., benefits), and discrimination concerns strongly influenced attitudes and intent for sickle cell trait screening among individuals identifying as Black/African American [33]. Parents strongly supported pediatric and newborn GT, even without available treatments, emphasizing the value of early diagnosis [36,43,48]. Discrete choice experiments indicated that both clinical utility and financial concerns shaped GT attitudes [40]. In hypothetical GT scenarios, framing of the decision affected opting for GT, yet there was high satisfaction and low regret for GT of both rare and common conditions [44]. A large longitudinal study within the 100,000 Genomes project revealed that participants with less knowledge of GS and less positive attitudes towards GS exhibited greater decisional regret 12–18 months later [50], underscoring the importance of high-quality decisions that are informed and aligned with values and preferences.

### 3.5. Mixed-Methods Studies (n = 3)

Three studies used mixed methods to capture quantitative data and explore it using qualitative inquiry [29,38,47]. Investigators leveraged different, complementary data sources to deepen insights and triangulate findings, providing a more nuanced understanding than a purely quantitative or qualitative approach alone. Ibisler et al. 2017 used surveys and open-ended questions to assess parental views on expanded newborn screening [29]. Nearly all parents supported GT for both treatable and untreatable conditions, citing preparedness and informed choice as key motivators. Schwartz and colleagues [38] combined validated measures (State-Trait Anxiety Inventory, Beck Depression Inventory) with qualitative interviews to elucidate how anxiety/depression relates to utilizing GT for susceptibility to genetic prion diseases. Having GT did not alleviate anxiety/depression, and many who tested positive chose to ignore their risk and had limited ongoing engagement with healthcare following GT. Gregersen et al. [47] conducted a medical chart review to capture health metrics/clinical outcomes, then re-contacted retinoblastoma survivors and invited them to receive genetic counseling. Investigators measured uptake and employed a qualitative researcher-developed survey to contextualize survivors’ experiences and attitudes related to genetic counseling/GT. Despite some uncertainty and emotional distress, survivors valued GT for informing them of their risk and disease management.

### 3.6. Promoters and Barriers to Genetic Testing

**Figure 3:**
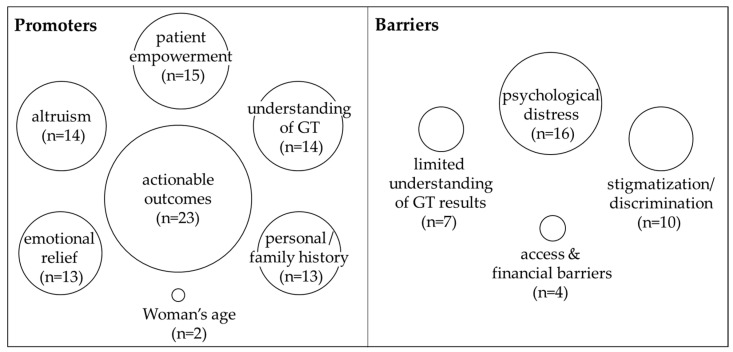
Constellation of thematic promoters and barriers to genetic testing. The size of each circle is proportional to the number of times the theme was noted across the 32 included articles.

Thematic analysis revealed both promoters and barriers to GT in the context of rare disorders. While all themes were important, certain themes were more frequent, suggesting they were prominent, yet not the sole psychosocial factors (Figure 3). Among the promoters, the most frequent theme was ‘actionable outcomes’ [22,23,25,26,31,34,35,36,37,38,39,40,41,42,43,45,47,48,50,51,52,53]. Across studies, people saw value in GT for informing reproductive planning, as well as medical and treatment decisions. ‘Altruism’ appeared frequently [22,23,24,25,31,34,37,42,44,45,46,47,49,53]. Genetic testing was seen as a way to benefit family and contribute to research, thereby potentially helping future generations. ‘Understanding of genetic testing’ was commonly noted as playing a crucial role in shaping attitudes [24,27,28,29,30,31,33,34,36,37,42,47,50,53]. Knowledge of GT and an understanding of the process contributed to a sense of confidence in deciding to undergo testing. ‘Patient empowerment’ was noted as a promoter [23,24,25,28,31,34,36,37,39,40,41,42,48,49,53]. Many individuals across studies expressed that GT provided them with greater control over their health. Similarly, ‘emotional relief’ was also common [22,23,25,31,35,37,38,41,42,45,47,49,51]. Individuals expressed a sense of comfort and relief in identifying or confirming a diagnosis or, conversely, ruling out health concerns. Having a ‘personal/family history’ or positive history of a rare disorder was a promoter [22,24,27,29,30,33,37,40,41,43,47,51,52]. People with a lived experience with a rare disorder were frequently in support of and advocated for testing. In studies of GT relating to reproductive decision-making, ‘woman’s age’ [24,32] had a role in the decision-making process.

Barriers were identified that complicated, discouraged, or prevented GT in the context of rare diseases. The most frequently cited concern was ‘psychological distress’ while awaiting results [23,24,26,28,31,34,35,38,39,42,45,46,47,49,52,53]. Across studies, uncertainty contributed to perceived emotional burden and anxiety. Concerns about ‘stigmatization/discrimination’ [22,23,28,35,38,44,46,49,52,53] were identified. Specifically, people expressed worry about potential implications for employment, insurance coverage, or relationships. ‘Limited understanding of results’ [24,26,28,37,38,39,52] contributed to hesitancy about testing decisions. For example, several studies reported that individuals had difficulty interpreting test results and/or were uncertain about what results would mean for their health. Additionally, ‘access and financial barriers’ [24,28,37,53] limited uptake of GT, particularly in cases when testing was not covered by insurance or was prohibitively expensive.

### 3.7. Lifespan Perspective on Genetic Testing Decisions

We considered that psychosocial factors may differ according to one’s stage in life. To capture differing lifespan perspectives, we sorted studies into life stage groups, then used thematic analysis to capture salient themes. The number of publications varied across life stages, ranging from 4–13. The studies examining the preconception stage [22,30,43,51] revealed the importance of ‘actionable results’ [22,43,51] and the critical role of a ‘personal/family history’ of the condition [22,30,43,51] as drivers. Additional positive aspects included ‘emotional relief’ [22,51], having an understanding of GT [30], and ‘altruism’ [22], while concerns about ‘stigmatization/discrimination’ [22,30] and ‘psychological distress while awaiting GT results’ [30] emerged as challenging aspects. In total, themes suggest that decision-making at this stage is largely driven by planning for the future.

Studies focusing on the prenatal stage [24,27,28,30,32,36,48,51,52] reported shared themes underscoring positive aspects of GT, including obtaining ‘actionable results’ [36,48,51,52] and ‘emotional relief’ (e.g., answers, diagnosis) [51], as well as ‘patient empowerment’ [24,28,48] and ‘altruism’ [24]. Having an ‘understanding of GT’ [24,27,28,30,36] and having a ‘personal/family history’ [24,27,30,51] were important moderating factors affecting decision-making. ‘Age’ [24,32] only appeared as a key decision-making theme in this life stage. Themes conveying negative emotional responses included ‘psychological distress while awaiting results’ [24,28,52], ‘limited understanding of results’ [24,28,52], and fears of ‘stigmatization/discrimination’ [28,52]. ‘Access and financial barriers’ [24,28] posed logistical obstacles to GT in the prenatal stage (Table 2).

The studies examining the pediatric stage [23,26,31,35,37,40,41,42,46,49,50] focused on GT decisions made for or by children and adolescents/young adults. Common themes included ‘emotional relief’ [23,31,35,37,41,42,49], ‘altruism’ [23,31,37,42,46,49], and ‘patient empowerment’ [23,31,37,40,41,42,49], reflecting how emotional experiences and relational dynamics shape testing decisions. As with earlier life stages, ‘understanding of GT’ [31,37,42,50], obtaining ‘actionable results’ [23,26,31,35,37,40,41,42,49,50], and having a ‘personal/family history’ [37,40,41] were important factors. ‘Psychological distress’ while awaiting results [23,26,31,35,42,46,49] and concerns about ‘stigmatization/discrimination’ [23,35,46,49] appeared across several studies. ‘Limited understanding of results’ [26,37,41] was challenging, and ‘access and financial barriers’ [37] was cited as a limiting factor for GT. Overall, study findings highlighted the range of informational, emotional, and contextual factors affecting decisions in the pediatric context (Table 2).

The studies examining personal GT in the adult stage of life [23,29,31,33,34,38,39,44,45,46,47,50,53] highlighted recurring themes of ‘understanding of GT’ [29,31,33,34,47,50,53], having a ‘personal/family history’ [29,33,47], ‘emotional relief’ (with diagnosis) [25,31,38,45,47], and obtaining ‘actionable results’ [25,31,34,38,39,45,47,50,53], as well as ‘altruism’ [25,31,34,44,45,46,47,53] and ‘patient empowerment’ [25,31,34,39,53]. Perceived challenges included ‘psychological distress’ while awaiting results [31,34,38,39,45,46,47,53], ‘limited understanding of results’ [38,39], and concerns about ‘stigma or discrimination’ [38,44,46,53]. As with earlier life stages, ‘access/financial barriers’ [53] limited GT.

### 3.8. Findings Mapped to the Theory of Planned Behavior

**Figure 4:**
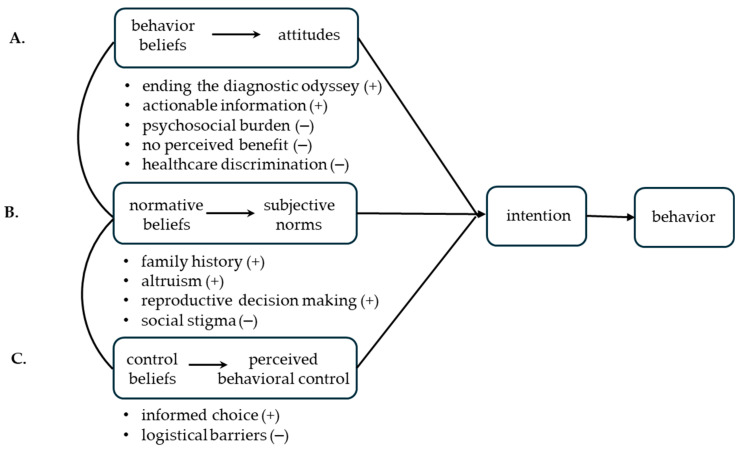
Findings mapped to the Theory of Planned Behavior (TPB). Promoters (+) and barriers (−) to genetic testing (GT) are depicted as bulleted points under the respective TPB component. (**A**) Behavior beliefs shape attitudes (i.e., perceived benefits or limitations). (**B**) Normative beliefs shape subjective norms (i.e., expectations of family/community/healthcare providers and cultural norms). (**C**) Control beliefs shape perceived behavioral control (i.e., agency, self-efficacy). (**A**–**C**) all shape intention, which precedes behavior. In this example, the ‘behavior’ is making a high-quality testing decision, one that is informed and aligned with values and preferences.

To inform future intervention development, we mapped synthesized findings onto the attitudes, subjective norms, and perceived behavioral control elements of the Theory of Planned Behavior (TPB) (Figure 4).

Attitudes reflect beliefs about the consequences of GT. In some instances, promoters and barriers represented opposite sides of the same topic. For example, the opportunity to ‘end the diagnostic odyssey’ (n = 16) [23,25,28,31,35,36,37,38,40,41,42,45,46,47,48,49] brought a sense of relief and closure with receiving a diagnosis and favored GT. Conversely, ‘psychosocial burden’ (n = 14) [23,30,31,34,35,36,38,39,40,42,46,47,49,52] representing the fear related to the revelation of an untreatable or life-shortening condition inhibited GT. Similarly, the opportunity to receive ‘actionable information’ (n = 24) [22,23,24,25,26,29,31,34,35,36,37,38,40,41,42,43,45,47,48,49,50,51,52,53] (i.e., results that inform medical decisions, treatment, and reproductive decisions) promoted the uptake of GT, while ‘no perceived benefit’ (n = 10) [30,33,37,38,39,41,42,45,46,52] discouraged uptake. Concerns about ‘healthcare discrimination’ (n = 10) [23,33,35,38,40,44,49,52,53], potentially losing employment or health insurance, contributed to a negative attitude about GT and posed a barrier for uptake.

Subjective norms relate to the social pressures (e.g., family, community, healthcare providers) and cultural expectations that can influence GT decision-making. Having an awareness of the impact of living with a rare disease via ‘family history’ (n = 18) [22,23,24,27,29,30,31,34,35,38,39,40,41,45,47,49,51,52] was cited as a type of family norm that motivated many to pursue GT. In the context of ‘reproductive decision-making’ (n = 19), the high value placed on a healthy family was a driver of preconception/prenatal GT decisions to inform family planning [22,24,28,29,30,34,35,38,39,41,42,43,46,47,48,49,51,52,53]. A norm of giving back emerged in ‘altruism’ (n = 6) [23,25,35,42,46,53] that was a motivator in several studies. Individuals reported a desire to have GT to contribute to research that would hopefully benefit future generations. In contrast, ‘social stigma’ (n = 10) [23,33,35,38,40,44,49,52,53] was a community norm and barrier; individuals were concerned about discrimination and stigmatization within cultures/communities that may view genetic conditions negatively.

Perceived behavioral control refers to a sense of agency and ability to act on their decision. Having an ‘informed choice’ (n = 18) [24,26,27,29,30,31,32,33,36,37,42,43,46,47,48,50,52,53] was a common promoter. People who understood the purpose, benefits, and limitations of GT were likely to undergo testing, underscoring the importance of access to genetic counseling. ‘Logistical barriers’ (n = 7) [24,26,28,33,37,46,53] posed obstacles. Structural aspects, including high cost of testing, inadequate insurance coverage, and geographic barriers to accessing genetic counseling, contributed to the lack of GT. Taken together, the promoters/barriers relating to attitudes, subjective norms, and perceived behavioral control represent targets for intervention development.

## 4. Discussion

Modern genetic testing (GT) is a powerful tool for shortening the ‘diagnostic odyssey’ for patients and families affected by rare and undiagnosed diseases [4,5,6]. Herein, we report a synthesis of current literature on the psychosocial factors related to GT in the context of rare disorders. While GT has rapidly expanded over the past decade, we did not see a parallel increase in articles examining GT decision-making over the past ten years. But for one exception, all identified studies were descriptive, and the majority (65%) of articles originated from Anglophone countries. Thus, much of what we know is descriptive and from high-income countries. While advances in next-generation sequencing and novel bioinformatic approaches have propelled rare disease discovery and fueled drug discovery efforts [54], our understanding of the psychosocial factors related to GT for rare diseases has not paralleled technologic growth. To realize the full potential of genomic discovery and GT for patients and families affected by rare diseases, understanding the human factor is a critical aspect of implementation.

### 4.1. Logistical/Structural Barriers to Genetic Testing

A consistent theme emerging from the included articles was the role of logistical barriers in accessing genetic counseling and GT. Two key structural obstacles emerged from the synthesis. First, financial barriers were a frequently cited barrier. Across studies, the high cost of GT/services and/or inadequate insurance coverage were problematic. Notably, financial barriers are often cited as a barrier to GT [55] in the context of common (i.e., Tier 1) genetic conditions, including hereditary cancers [54,56] and familial hypercholesterolemia [57]. Thus, financial barriers appear to be a common, shared experience for patients/families, regardless of the prevalence of a given genetic disease. Such logistical, structural obstacles require broader systemic and policy changes. A recent systematic review on payer perspectives on GT summarized challenges and opportunities for GT coverage [58]. Understanding stakeholder perspectives will be critical for finding solutions that effectively align both conceptual frameworks for genomic healthcare (e.g., weighing personal utility of GT that includes behavioral, affective, cognitive, and social outcomes) and evidence demonstrating the value of GT.

Geography was an important logistical barrier. Specifically, the distance from medical services and specialized/genetic care, as well as the lack of remote counseling/testing options, posed significant challenges. Geography is a long-acknowledged challenge to the care of rare diseases, and there is growing recognition of the geographic inequities in genomic healthcare [59]. Decision aids have been demonstrated to be effective for extending the reach of healthcare services for patients and families [60]. There has been growing interest in using remote genetic counseling [61]. The trend appeared to accelerate during the COVID-19 pandemic [62] and is growing internationally to meet the burgeoning need for genetic counseling [63]. Remote GT decisional support has gained traction in the context of hereditary breast and ovarian cancers (HBOC) [11], and tools have been piloted for both diagnostic and cascade screening purposes [10,64]. The emergence of artificial intelligence (AI) has opened new avenues for extending the reach of genetic counseling/decisional support [65], including chatbots leveraging large language models [66]. Such developments hold promise as future directions for surmounting geographic barriers for rare diseases.

Interestingly, an interventional study reported herein [44] randomized participants to one of two hypothetical GT scenarios—either a rare, life-altering (congenital hypogonadotropic hypogonadism) or a common, life-threatening genetic condition (HBOC)—to examine the effect of behavioral nudges on GT decision-making. Interestingly, the study found striking similarities in decision cognitions between the hypothetical GT scenarios supporting the notion of an overlapping “common:rare” paradigm and suggested that findings could be used to support a modular approach to decisional support, regardless of disease prevalence or lethality. Subsequently, a group of international investigators adopted the “common:rare” paradigm to create an empirically based framework (ACCESS: Advocating, Coping, Communication, cascadE Screening, and Surveillance) to address disparities in genomic healthcare [16].

### 4.2. Similarities in Psychosocial Factors Between Rare and Common (Tier 1) Genetic Diseases

Decisional support for GT is multifaceted, nuanced, and can be considered a complex intervention. Current guidance on complex interventions underscores the importance of using theory to guide intervention development [14]. The Theory of Planned Behavior is a widely used psychological framework employed to help predict and understand human behavior and decision-making [15]. To inform future intervention development (i.e., decisional support), we synthesized the promoters and barriers to GT for rare diseases and mapped those factors amenable to intervention onto the Theory of Planned Behavior (i.e., ‘attitudes’, ‘subjective norms’, and ‘perceived behavioral control’). Interestingly, a number of themes emerging from the synthesis overlapped with findings from systematic reviews examining informational needs [12], psychosocial aspects [56,67], and family communication [68] related to GT for common (i.e., Tier 1) inherited cancers (i.e., HBOC, Lynch Syndrome). Attitudes affecting rare disease GT included the positive aspects of potentially ending the diagnostic odyssey and actionable findings that could inform care and treatment. Such themes parallel the value placed on obtaining information to support informed care/treatment in the context of HBOC [12,67]. For rare disease patients, uncertainty, anxiety/distress, and fear of discrimination posed barriers to GT, findings that were also cited in Tier 1 genetic conditions [56,67].

We identified several ‘norms’ that also shaped decision-making for rare disease GT, including understanding the impact rare disease has on the family, informed reproductive decision-making, and altruism. A 2024 review examining psychosocial factors affecting the uptake of cascade carrier screening found similar themes, including understanding of genetic/cancer consequences, family closeness/support, and a sense of responsibility promoting GT and intrafamilial communication [67]. In the context of HBOC, reproductive decisions are similarly a key factor in GT decision-making [69,70]. In studies of rare diseases, community/cultural norms resulting in social stigma inhibited GT. In studies of Tier 1 conditions, fear of stigma/discrimination limited GT [56], as did strained family dynamics and negative family reactions to GT [67].

Perceived behavioral control (i.e., agency) is a strong determinant of intention and subsequent behavior. In the rare disease context, being able to make an informed choice was a promoter of agency in pursuing GT. Studies in HBOC identify having information, decisional empowerment, and self-efficacy as drivers promoting GT [12,67]. A 2025 systematic review on decision-making in the context of pediatric GT identified key emotional, relational, and contextual factors affecting parent/guardian decision-making for diagnostic and predictive GT [71]. The work extends an empirical decision-making model for pediatric genomics [9]. Interestingly, our mapping to the TPB bears similarities with such works, as we similarly identified key cognitive, affective, and relational (norms) psychosocial factors that heavily influence decision-making for GT. Taken together, these findings can help propel theory-informed interventions supporting patients and families in GT decisions.

### 4.3. Patient and Public Involvement Insights

The patient advocate collaborator provided a number of key insights on the synthesized findings. In terms of challenges, he noted that patients may not understand what GT can realistically achieve (i.e., lack of understanding of GT). Further, many patients expect to receive definitive results and are unaware that inconclusive results (i.e., variants of uncertain significance) are possible or even probable. He also emphasized that some patients may rely on outdated test results from years ago, not recognizing that advances in sequencing and expanded genetic knowledge could inform their case. Importantly, many patients are deeply concerned about passing their condition to future generations, yet many patients/families are unable to access genetic counseling that could provide clarity about inheritance risks. He shared that patients typically have a limited understanding of genetics. For example, in congenital hypogonadotropic hypogonadism, an individual may have a change in a gene associated with the condition, which may not result in physical symptoms (i.e., incomplete penetrance, variable expressivity). He added that when patients do receive GT results, they often lack sufficient support to interpret findings.

The patient advocate commented on several positive aspects of GT and emphasized that GT can be empowering. Testing can help end the ‘diagnostic odyssey’, offer closure, and provide a deeper understanding of an individual’s disorder, even when treatment remains unchanged. Notably, patient attitudes towards GT in the context of research vary widely. Some patients are eager to provide samples for any research, while others participate only when they perceive a direct personal benefit. He added that patients may not fully appreciate the importance of altruism related to GT research. Specifically, while research may not alter their treatment or patient journey, participation may help improve outcomes for future generations—a powerful motivating factor. Lastly, the patient advocate was excited by the prospect of developing AI systems with specialized rare disease ‘knowledge’ to bridge knowledge gaps, help patients access condition-specific information, and support better-informed decision-making about GT and its implications. Moreover, such digital solutions may enable a more global reach, as translation capabilities could be embedded to help bridge language barriers for non-English-speaking patients/families.

### 4.4. Future Directions for Person-Centered Genetic Testing Decisional Support

Together, these findings indicate a number of overlapping promoters and barriers to GT between rare and common genetic disorders. Such findings favor the possibility of a modular, asynchronous, web-based approach to GT decisional support [44]. For example, content responding to a core set of psychosocial factors (overlapping between common and rare) could be delivered as a central component. Disease-specific content could be added to create a tailored, modular approach. Such digital solutions could help scale and extend the reach of decisional support to bolster high-quality decisions (i.e., informed and aligned with values and preferences). An asynchronous, web-based approach (with or without AI enhancements) could help meet the growing demand for pre-test genetic counseling, as there is currently a significant shortage of genetic counselors [72,73]. It is worthwhile to note that digital solutions should complement the healthcare provider’s role rather than serve as an in silico replacement. Combined with results from studies of Tier 1 conditions, our synthesis of findings underscores the importance of healthcare providers’ effective communication, recommendation, and shared decision-making for the uptake of GT [56,67,68].

### 4.5. Strengths and Limitations

Relative strengths of the investigation include the comprehensive, systematic literature search, use of structured search terms, a rigorous dual review process, and using the well-established Arksey and O’Malley framework. Another relative strength of the study is the lifespan perspective that was employed. Our synthesis of findings from the identified articles revealed a relatively high degree of agreement in the barriers/promoters of GT across the life cycle. However, it is worthwhile to note that these stable, shared factors may be experienced, interpreted, and weighted differently by an individual, depending on life cycle context. As such, a developmental lens may be useful, as an individual’s specific stage in life and corresponding relational/familial roles are important considerations. Mapping the synthesis to the TPB may be considered a strength, as it may serve as a basis for developing theory-informed interventions in the future.

There are a number of limitations that merit noting. First, there are an estimated 7000 rare diseases, and despite using a rigorous systematic search and review process, it is possible that we did not identify all articles examining GT in the context of rare diseases. As we only included articles published in English, we recognize the Anglophone bias and acknowledge that there may be articles published in other languages that were not included. Findings from the quantitative and mixed-methods studies should be interpreted with caution, as relatively few studies employed validated instruments/tools. This likely reflects the fact that generic tools do not accurately capture or measure the constructs that are salient to rare diseases. The limited number (n = 3) of mixed-methods studies points to an important future direction for the field. Mixed methods have the potential to capture the complexity of patient/family experiences, decision-making, and psychosocial impacts, providing a deeper understanding and more nuanced insights than single method approaches. We recognize that psychosocial factors related to GT decision-making may differ and vary according to life stage, and we grouped studies accordingly. However, there were relatively few studies in each period from preconception through adulthood. The limited sample size precluded creating robust lifespan-specific recommendations. The comparison with common (Tier 1) conditions in the discussion is based on a number of recent systematic reviews. It is worthwhile to note that we did not conduct a systematic literature search for the “common: rare” comparison.

## 5. Conclusions

The synthesis of findings on GT for rare diseases identified logistical/structural barriers to GT (i.e., finances, geography) and psychosocial factors amenable to intervention. We identified a number of psychosocial factors that overlap between rare and common (Tier 1) genetic diseases. Findings represent targets for scalable, theory-informed interventions to support high-quality GT decisions, regardless of disease prevalence (i.e., rare or common). Technology and AI provide novel avenues to extend the reach of decisional support, GT, and care for patients and families affected by rare diseases.

## Figures and Tables

**Table 1:** Types of genetic testing examined in identified articles (n = 32).

Type	Number	References
diagnostic	16	[23,26,27,29,34,35,36,38,39,41,45,48,51,52]
predictive	14	[22,23,24,25,31,32,37,40,41,42,45,46,47,49,50,53]
carrier screening	5	[24,26,30,33,34]
hypothetical testing scenarios	2	[39,44]

**Table 2:** Lifespan perspective on barriers and promoters of genetic testing (GT) for rare diseases.

	Preconception (n = 4)	Prenatal (n = 9)	Pediatric (n = 11)	Adult (n = 13)
**Promoters**				
actionable results	[22,43,53]	[36,43,48,53]	[23,26,31,35,37,40,41,42,49,50]	[25,31,34,38,39,45,47,50,52]
personal/family history	[22,30,43,53]	[24,27,30,43,53]	[37,40,41]	[29,33,47]
emotional relief	[22,43]	[53]	[23,31,35,37,41,42,49]	[25,31,38,45,47]
altruism	[22]	[24]	[23,31,37,42,46,49]	[25,31,38,44,45,46,47,52]
understanding of GT	[30]	[24,27,28,30,36]	[31,37,42,50]	[29,31,33,34,47,50,52]
patient empowerment	-	[24,28,48]	[23,31,37,40,41,42,49]	[25,31,34,39,52]
woman’s age	-	[24,32]	-	-
**Barriers**				
stigmatization/ discrimination	[22,30]	[28,30,51]	[23,35,46,49]	[38,44,46,52]
psychological distress	[30]	[24,28,30,51]	[23,26,31,35,42,46,49]	[31,34,38,39,46,47,52]
access and financial barriers	-	[24,28]	[26,37]	[52]
limited under- standing of results	-	[24,28,51]	[26,37,41]	[38,39,46]

Overall, decision-making in adulthood is shaped by a balance of clinical relevance, emotional drivers, and perceived social consequences.

## Data Availability

The full data extraction table is provided in Table S1.

## Supplementary Materials

The following supporting information can be downloaded at: https://www.mdpi.com/article/10.3390/genes16060614/s1, Table S1: Data Extraction Table [22,23,24,25,26,27,28,29,30,31,32,33,34,35,36,37,38,39,40,41,42,43,44,45,46,47,48,49,50,51,52,53] (Included studies data extraction table).

## Abbreviations

The following abbreviations are used in this manuscript:

AI	artificial intelligence
GS	genomics sequencing
GT	genetic testing
HBOC	hereditary breast and ovarian cancer syndrome
NORD	National Organization of Rare Disorders
TPB	Theory of Planned Behavior
U.K.	United Kingdom
U.S.	United States

## Appendix A

### Appendix A.1. Search Terms

We used the search term ‘decision-making’, as it relates closely to psychosocial factors related to genetic testing.

PubMed

((decision-making[MeSH Terms]) AND (((genetic testing[MeSH Terms]) AND (rare disease[MeSH Terms])) OR ((genetic testing[MeSH Terms]) AND (genetic disease, rare[MeSH Terms]))) AND (y_10[Filter])) OR ((decision-making[MeSH Terms]) AND (((genetic testing[MeSH Terms]) AND (inborn disease[MeSH Terms])) OR ((genetic testing[MeSH Terms]) AND (genetic disease, inborn[MeSH Terms]))) AND (y_10[Filter]))

(((decision-making[MeSH Terms]) AND (((genetic testing[MeSH Terms]) AND (rare disease[MeSH Terms])) OR ((genetic testing[MeSH Terms]) AND (genetic disease, rare[MeSH Terms]))) AND (y_10[Filter])) OR ((decision-making[MeSH Terms]) AND (((genetic testing[MeSH Terms]) AND (inborn disease[MeSH Terms])) OR ((genetic testing[MeSH Terms]) AND (genetic disease, inborn[MeSH Terms]))) AND (y_10[Filter])) AND (y_10[Filter])) AND (“patient attitude*” or “patient belief*” or “patient view*” or “patient feeling*” or “patient hope*” or “patient confidence” or “psychological stress” or “patient fear*” or “patient anxiety” or “patient apprehension”) Filters: in the last 10 years

(((decision-making[MeSH Terms]) AND (((genetic testing[MeSH Terms]) AND (rare disease[MeSH Terms])) OR ((genetic testing[MeSH Terms]) AND (genetic disease, rare[MeSH Terms]))) AND (y_10[Filter])) OR ((decision-making[MeSH Terms]) AND (((genetic testing[MeSH Terms]) AND (inborn disease[MeSH Terms])) OR ((genetic testing[MeSH Terms]) AND (genetic disease, inborn[MeSH Terms]))) AND (y_10[Filter])) AND (y_10[Filter])) AND (“patient influence*” or “patient culture*” or “patient perception*” or “patient experience*” or “counsel*”) Filters: in the last 10 years

Embase

((‘decision making’/exp OR ‘decision making’) AND ((‘genetic testing’/exp OR ‘genetic testing’ OR ((‘genetic’/exp OR genetic) AND (‘testing’/exp OR testing))) AND (‘rare disease’/exp OR ‘rare disease’ OR (rare AND (‘disease’/exp OR disease))) OR ((‘genetic testing’/exp OR ‘genetic testing’ OR ((‘genetic’/exp OR genetic) AND (‘testing’/exp OR testing))) AND (‘genetic disease, rare’ OR ((‘genetic’/exp OR genetic) AND (‘disease,’/exp OR disease,) AND rare)))) OR ((‘decision making’/exp OR ‘decision making’) AND ((‘genetic testing’/exp OR ‘genetic testing’ OR ((‘genetic’/exp OR genetic) AND (‘testing’/exp OR testing))) AND (‘inborn disease’ OR (inborn AND (‘disease’/exp OR disease))) OR ((‘genetic testing’/exp OR ‘genetic testing’ OR ((‘genetic’/exp OR genetic) AND (‘testing’/exp OR testing))) AND (‘genetic disease, inborn’ OR ((‘genetic’/exp OR genetic) AND (‘disease,’/exp OR disease,) AND inborn)))))) AND [2014–2025]/py

((‘decision making’/exp OR ‘decision making’) AND ((‘genetic testing’/exp OR ‘genetic testing’ OR ((‘genetic’/exp OR genetic) AND (‘testing’/exp OR testing))) AND (‘rare disease’/exp OR ‘rare disease’ OR (rare AND (‘disease’/exp OR disease))) OR ((‘genetic testing’/exp OR ‘genetic testing’ OR ((‘genetic’/exp OR genetic) AND (‘testing’/exp OR testing))) AND (‘genetic disease, rare’ OR ((‘genetic’/exp OR genetic) AND (‘disease,’/exp OR disease,) AND rare)))) OR ((‘decision making’/exp OR ‘decision making’) AND ((‘genetic testing’/exp OR ‘genetic testing’ OR ((‘genetic’/exp OR genetic) AND (‘testing’/exp OR testing))) AND (‘inborn disease’ OR (inborn AND (‘disease’/exp OR disease))) OR ((‘genetic testing’/exp OR ‘genetic testing’ OR ((‘genetic’/exp OR genetic) AND (‘testing’/exp OR testing))) AND (‘genetic disease, inborn’ OR ((‘genetic’/exp OR genetic) AND (‘disease,’/exp OR disease,) AND inborn)))))) AND (‘patient attitude*’ OR ‘patient belief*’ OR ‘patient view*’ OR ‘patient feeling*’ OR ‘patient hope*’ OR ‘patient confidence’/exp OR ‘patient confidence’ OR ‘psychological stress’/exp OR ‘psychological stress’ OR ‘patient fear*’ OR ‘patient anxiety’ OR ‘patient apprehension’) AND [2014–2025]/py

((‘decision making’/exp OR ‘decision making’) AND ((‘genetic testing’/exp OR ‘genetic testing’ OR ((‘genetic’/exp OR genetic) AND (‘testing’/exp OR testing))) AND (‘rare disease’/exp OR ‘rare disease’ OR (rare AND (‘disease’/exp OR disease))) OR ((‘genetic testing’/exp OR ‘genetic testing’ OR ((‘genetic’/exp OR genetic) AND (‘testing’/exp OR testing))) AND (‘genetic disease, rare’ OR ((‘genetic’/exp OR genetic) AND (‘disease,’/exp OR disease,) AND rare)))) OR ((‘decision making’/exp OR ‘decision making’) AND ((‘genetic testing’/exp OR ‘genetic testing’ OR ((‘genetic’/exp OR genetic) AND (‘testing’/exp OR testing))) AND (‘inborn disease’ OR (inborn AND (‘disease’/exp OR disease))) OR ((‘genetic testing’/exp OR ‘genetic testing’ OR ((‘genetic’/exp OR genetic) AND (‘testing’/exp OR testing))) AND (‘genetic disease, inborn’ OR ((‘genetic’/exp OR genetic) AND (‘disease,’/exp OR disease,) AND inborn)))))) AND (‘patient influence*’ OR ‘patient culture*’ OR ‘patient perception*’ OR ‘patient experience*’ OR ‘counsel*’) AND [2014–2025]/py

Web of Science, Cochrane, Sociology Abstracts, and Journal of Biomedical Informatics

((decision-making) AND (((genetic testing) AND (rare disease)) OR ((genetic testing) AND (genetic disease, rare)))) OR ((decision-making) AND (((genetic testing) AND (inborn disease)) OR ((genetic testing) AND (genetic disease, inborn))))

(((decision-making) AND (((genetic testing) AND (rare disease)) OR ((genetic testing) AND (genetic disease, rare)))) OR ((decision-making) AND (((genetic testing) AND (inborn disease)) OR ((genetic testing) AND (genetic disease, inborn))))) AND (“patient attitude*” or “patient belief*” or “patient view*” or “patient feeling*” or “patient hope*” or “patient confidence” or “psychological stress” or “patient fear*” or “patient anxiety” or “patient apprehension”)

(((decision-making) AND (((genetic testing) AND (rare disease)) OR ((genetic testing) AND (genetic disease, rare)))) OR ((decision-making) AND (((genetic testing) AND (inborn disease)) OR ((genetic testing) AND (genetic disease, inborn))))) AND (“patient influence*” or “patient culture*” or “patient perception*” or “patient experience*” or “counsel*”)
